# Association between serum 25-hydroxyvitamin D levels and thyroid function alterations in hospitalized adults: a retrospective cross-sectional study

**DOI:** 10.3389/fendo.2026.1959544

**Published:** 2026-10-14

**Authors:** Hantian Zheng, Meizhen Li, Dingnan Lin, Lin Yang, Zhiping Yang, Ting Wu, Yijie Li, Shaojie Liu

**Affiliations:** 1 Department of Clinical Nutrition, First Affiliated Hospital of Xiamen University, School of Medicine, Xiamen University, Xiamen, China; 2 School of Public Health, Xiamen University, Xiamen, China; 3 School of Clinical Medicine, Fujian Medical University, Fuzhou, China; 4 Department of Epidemiology and Health Statistics, School of Public Health, Fujian Medical University, Fuzhou, China

**Keywords:** cross-sectional study, hospitalized adults, serum 25-hydroxyvitamin D, thyroid function alterations, thyroid hormones

## Abstract

**Background:**

Thyroid function abnormalities and low vitamin D status are common in hospitalized adults, but evidence on their association remains limited and inconsistent.

**Methods:**

We conducted a retrospective cross-sectional study of 225 adult inpatients between January 2020 and January 2025. Serum 25-hydroxyvitamin D [25(OH)D] and thyroid function indicators were measured during the same admission. Thyroid function alterations were defined as any TSH, FT3, or FT4 value outside the corresponding laboratory reference range. Associations of 25(OH)D with thyroid-related indicators and thyroid function alterations were evaluated using multivariable linear and logistic regression models. Sensitivity, subgroup, and restricted cubic spline analyses were also performed.

**Results:**

Among 225 participants, 91 (40.4%) had thyroid function alterations. Median serum 25(OH)D was lower in participants with thyroid function alterations than in those with normal thyroid function (18.00 vs 21.40 ng/mL). In the fully adjusted model, ln[25(OH)D] was positively associated with FT4, FT3, T3, and T4 and inversely associated with TSH. Continuous ln[25(OH)D] was inversely associated with the odds of thyroid function alterations in Models A–C (Model C: OR 0.566, 95% CI 0.317–0.988), but the association was attenuated after further adjustment for TP and CRP in Model D (OR 0.569, 95% CI 0.309–1.021). After excluding participants with isolated low FT3, the inverse association remained similar in direction but was not statistically significant in the fully adjusted model. Significant interactions were observed by both sex and age group, with inverse associations observed in men and participants aged ≥65 years. Restricted cubic spline analysis showed no significant overall or nonlinear association.

**Conclusions:**

Serum 25(OH)D was associated with multiple thyroid function indicators in hospitalized adults, whereas its inverse association with thyroid function alterations was attenuated after further adjustment for TP and CRP. Prospective studies are needed to clarify the temporal direction and clinical significance of these associations.

## 1 Introduction

Thyroid hormones are important regulators of energy homeostasis, cardiovascular function, and human brain activity (1–3). Even mild thyroid dysfunction has been associated with adverse clinical outcomes (4), including arrhythmias (5), heart failure (5), skeletal fragility (6), and poorer health-related quality of life (7). In hospitalized patients, thyroid function abnormalities are common, but their interpretation may be complicated by nonthyroidal illness syndrome (NTIS) and other illness related changes (8–10). Acute illness, systemic inflammation, physiological stress, medication exposure, and nutritional disturbances may all affect the hypothalamic–pituitary–thyroid axis (8–11). Among these factors, nutritional factors are particularly relevant. For example, iodine is indispensable for thyroid hormone synthesis, and selenium is required for thyroid hormone metabolism and antioxidant defense within thyroid tissue (12, 13). Moreover, an increasing number of studies have begun to focus on the possible impact of vitamin D on thyroid function.

Vitamin D status, usually assessed by serum 25-hydroxyvitamin D [25(OH)D], has been proposed as a potentially modifiable factor related to thyroid function (14). Beyond its classical role in calcium-phosphate homeostasis and skeletal health, vitamin D receptor and vitamin D-metabolizing enzymes have been identified in thyroid tissue and immune cells (15, 16). This suggests that vitamin D may influence thyroid homeostasis through both endocrine and immune pathways. Previous human studies have explored the associations of 25(OH)D with thyroid function abnormalities and thyroid function indicators, but the findings have been inconsistent. Some observational studies reported that higher 25(OH)D levels were associated with lower TSH levels or lower risk of hypothyroidism (17, 18). In contrast, other studies did not observe significant associations between serum 25(OH)D and TSH, FT3, or FT4 (14, 19, 20). Randomized trials have also shown inconsistent effects of vitamin D supplementation on TSH and thyroid hormones (21). Overall, existing evidence remains heterogeneous and has been derived mainly from general populations. Evidence in hospitalized adults is still limited. Therefore, further investigation in inpatient populations is warranted.

In this study, we utilized electronic medical records from hospitalized adults in whom both serum 25(OH)D levels and thyroid function tests were measured during the same admission. Our aim was to explore the associations of serum 25(OH)D with thyroid function alterations and multiple thyroid-related indicators.

## 2 Materials and methods

### 2.1 Study population

This retrospective cross-sectional study was based on electronic medical records from the First Affiliated Hospital of Xiamen University, Fujian, China. We identified all adult inpatients between January 2020 and January 2025 who had serum 25(OH)D and complete thyroid function test results measured during the same admission. When a patient had more than one eligible hospitalization, only the first eligible admission was included to avoid duplication and potential interference from treatment during hospitalization.

We excluded patients who met any of the following criteria: 1) age younger than 18 years; 2) a history of thyroid cancer, thyroidectomy, or radioactive iodine therapy; 3) receipt of thyroid hormone replacement, antithyroid drugs, amiodarone, lithium, systemic glucocorticoids, or other medications known to markedly affect thyroid function or vitamin D metabolism; 4) pregnancy or lactation; 5) known pituitary or hypothalamic disease, adrenal insufficiency, or other major endocrine disorders affecting the hypothalamic–pituitary–thyroid axis; and 6) missing exposure or outcome data after data cleaning. The final analytic sample consisted of 225 patients. The study was approved by the institutional ethics committee of the First Affiliated Hospital of Xiamen University (approval No. [2025] KYLSZ(209)), which waived informed consent because only anonymized retrospective data were used.

### 2.2 Definition of thyroid function alterations

Thyroid function indicators included serum free triiodothyronine (FT3), free thyroxine (FT4), total triiodothyronine (T3), total thyroxine (T4), and thyroid-stimulating hormone (TSH), together with thyroid autoantibodies [thyroid peroxidase antibody (TPOAb), thyroglobulin antibody (TGAb), and thyrotropin receptor antibody (TRAb)]. Serum TSH, FT4, FT3, T3, T4, TPOAb, TGAb and TRAb were measured in the hospital central laboratory using standardized automated immunoassays. In addition, we quantified central sensitivity and resistance of the hypothalamic–pituitary–thyroid feedback using three established indices derived from FT4 and TSH: the thyrotroph T4 resistance index (TT4RI = FT4 × TSH), the TSH index (TSHI = ln[TSH] + 0.1345 × FT4) proposed by Jostel et al. in 2009 to estimate thyrotroph function beyond raw TSH, and the thyroid feedback quantile-based index (TFQI) proposed by Laclaustra et al. in 2019 to robustly capture the FT4–TSH set point in population studies (TFQI = CDF(FT4) − [1 − CDF(TSH)]) (22, 23).

The laboratory reference ranges were 0.27–4.20 mIU/L for TSH, 12–22 pmol/L for FT4, and 3.1–6.8 pmol/L for FT3. For the primary analysis, thyroid function status was defined according to the values of TSH, FT4, and FT3 within their respective laboratory reference ranges. Normal thyroid function was defined as TSH, FT4, and FT3 all being within the corresponding reference ranges. Thyroid function alterations were defined as any value of TSH, FT4, or FT3 falling outside its corresponding reference range. This prespecified operational definition was used to identify biochemical alterations in thyroid function and was not intended to establish an etiologic diagnosis of primary thyroid disease. Participants with thyroid function alterations were further categorized according to the combination of TSH, FT4, and FT3 results into subclinical hypothyroidism, clinical hypothyroidism, subclinical hyperthyroidism, and clinical hyperthyroidism based on established biochemical patterns (24, 25). Isolated low FT3 and other discordant abnormalities were recorded separately because such patterns may occur in hospitalized patients with nonthyroidal illness (10).

### 2.3 Assessment of vitamin D status

Vitamin D status is commonly assessed by measuring circulating serum 25(OH)D (26). Serum 25(OH)D was measured in fasting venous blood by chemiluminescent immunoassay in the same central laboratory and was reported in ng/mL. In the main models, 25(OH)D was analyzed as a continuous variable after natural logarithmic transformation to reduce skewness. For categorical analyses, serum 25(OH)D was classified into <20, 20–<30, and ≥30 ng/mL according to commonly used clinical thresholds (26), with ≥30 ng/mL as the reference category. For the restricted cubic spline analysis, serum 25(OH)D was modeled as a continuous variable on its original scale.

### 2.4 Covariates

We considered covariates that were plausibly related to both serum 25(OH)D levels and thyroid function. Demographic factors included age and sex. Body mass index (BMI, kg/m²) was calculated from measured weight and height at admission. Smoking status was classified as ever or never, and season of blood draw was grouped into high-sunlight (May–November) and low-sunlight (December–April) periods. Laboratory markers included total protein (TP) and C-reactive protein (CRP). Missing covariates were imputed using multivariate imputation by chained equations (MICE) implemented in the R package mice (27). Estimates were pooled across imputed datasets using Rubin’s rules (28).

### 2.5 Statistical analysis

All analyses were conducted using R (version 4.5.1). A two-sided P value < 0.05 was considered statistically significant. For analyses involving multiple thyroid-related outcomes, the Benjamini–Hochberg method was applied within each adjustment model and analysis family, with q < 0.05 considered statistically significant. Baseline characteristics were summarized as medians with interquartile ranges for continuous variables and counts with percentages for categorical variables. Continuous variables were compared using the Wilcoxon rank-sum test, and categorical variables were compared using the Pearson χ² test. Associations of serum 25(OH)D with thyroid function indicators were evaluated using multivariable linear regression, while logistic regression was used to evaluate the odds of thyroid function alterations. FT4, FT3, TSH, T3, T4, TPOAb, TGAb, and TRAb were natural log-transformed before linear regression. Serum 25(OH)D was analyzed as a natural log-transformed continuous variable and as a categorical variable using <20, 20–<30, and ≥30 ng/mL, with ≥30 ng/mL as the reference group. Four adjustment models were constructed. Model A was unadjusted. Model B adjusted for age, sex, and BMI. Model C further adjusted for smoking status and season of blood draw. Model D further adjusted for TP and CRP.

As a sensitivity analysis, the logistic regression models were repeated after excluding participants with isolated low FT3, a biochemical pattern that may be compatible with NTIS. Subgroup analyses were then performed by sex and age (<65 and ≥65 years). Models were adjusted according to Model D, excluding the corresponding stratification variable, and interactions were tested using product terms. Restricted cubic spline logistic regression was performed to explore the dose-response relationship between serum 25(OH)D and thyroid function alterations. Serum 25(OH)D was modeled on its original scale using four knots at the 5th, 35th, 65th, and 95th percentiles, with the median concentration (20.0 ng/mL) as the reference. The spline model was adjusted according to Model D. Finally, exploratory LASSO penalized logistic regression with 10-fold cross-validation was performed using age, sex, BMI, season, smoking status, fasting plasma glucose (GLU), TP, CRP, and continuous 25(OH)D as candidate predictors. Discriminative performance was assessed using the cross-validated AUROC.

## 3 Results

### 3.1 Baseline characteristics

The baseline characteristics of study participants were shown in **Table 1**. A total of 225 adult inpatients were included in the analysis. Principal diagnosis information was available for 213 participants (94.7%), and the distribution of major disease categories is shown in **Supplementary Table S1**. Among all participants, 134 (59.6%) had normal thyroid function and 91 (40.4%) had thyroid function alterations. Among the 91 participants with thyroid function alterations, the most common biochemical pattern was subclinical hypothyroidism, followed by clinical hypothyroidism and isolated low FT3. Detailed biochemical patterns are shown in **Supplementary Table S2**.

**Table 1:** Baseline characteristics of study participants stratified by thyroid function status (*n* = 225).

Demographic characteristics	Normal thyroid function (n = 134)	Thyroid function alterations (n = 91)	*P-value*
Age, years	65.00 (46.50, 77.75)	67.00 (52.00, 81.50)	0.434
Sex			0.610
Male	56 (41.8%)	42 (46.2%)	
Female	78 (58.2%)	49 (53.8%)	
Marital status			0.217
Married	119 (88.8%)	86 (94.5%)	
Unmarried	15 (11.2%)	5 (5.5%)	
BMI (kg/m²)	22.77 (20.57, 25.08)	22.77 (20.09, 25.24)	0.829
Length of hospital stay, days	14.00 (8.00, 26.00)	13.00 (8.00, 39.00)	0.318
Season			0.062
High sunlight	94 (70.1%)	52 (57.1%)	
Low sunlight	40 (29.9%)	39 (42.9%)	
Smoking			0.999
Ever	22 (16.4%)	15 (16.5%)	
Never	112 (83.6%)	76 (83.5%)	
Serum 25(OH)D			
25(OH)D level (ng/mL)	21.40 (15.85, 26.53)	18.00 (13.25, 25.15)	0.028
25(OH)D category			0.236
<20 ng/mL	61 (45.5%)	51 (56.0%)	
20–<30 ng/mL	56 (41.8%)	33 (36.3%)	
≥30 ng/mL	17 (12.7%)	7 (7.7%)	
FT3 (pmol/L)	4.45 (3.90, 4.90)	3.81 (2.93, 4.51)	<0.001
FT4 (pmol/L)	16.36 (14.77, 17.94)	13.99 (11.61, 16.32)	<0.001
T3 (nmol/L)	1.52 (1.19, 1.72)	1.29 (0.97, 1.63)	0.007
T4 (nmol/L)	95.6 (78.7, 116.0)	84.0 (62.6, 96.6)	<0.001
TSH (mIU/L)	1.88 (1.15, 2.69)	4.31 (0.68, 6.18)	0.003
TPOAb (IU/mL)	3.79 (1.50, 31.07)	4.93 (1.83, 55.95)	0.156
TGAb (IU/mL)	13.00 (4.69, 64.42)	18.60 (6.99, 91.10)	0.178
TRAb (IU/L)	0.25 (0.25, 0.33)	0.30 (0.25, 0.73)	<0.001
AST (U/L)	20.90 (16.82, 27.90)	21.60 (17.25, 27.20)	0.583
TP (g/L)	69.15 (63.75, 72.40)	65.60 (59.35, 71.90)	0.023
GLU (mmol/L)	5.04 (4.49, 5.57)	5.12 (4.62, 6.46)	0.113
CRP (mg/L)	1.10 (0.50, 7.35)	4.94 (0.82, 29.28)	<0.001

Data were shown as median (IQR) or N (%). Abbreviations: BMI, body mass index; 25(OH)D, 25-hydroxyvitamin D; FT3, free triiodothyronine; FT4, free thyroxine; TSH, thyroid-stimulating hormone; T3, triiodothyronine; T4, thyroxine; TPOAb, thyroid peroxidase antibody; TGAb, thyroglobulin antibody; TRAb, thyrotropin receptor antibody; AST, aspartate aminotransferase; TP, total protein; GLU, fasting plasma glucose; CRP, C-reactive protein.

The two groups were similar in age, sex, marital status, BMI, length of hospital stay, season, smoking status, AST, and GLU. The median age was 65.0 years in the normal thyroid function group and 67.0 years in the thyroid function alterations group. Serum 25(OH)D levels were lower in participants with thyroid function alterations than in those with normal thyroid function, with median (IQR) values of 18.00 (13.25–25.15) and 21.40 (15.85–26.53) ng/mL, respectively (*P* = 0.028). The distribution of the three 25(OH)D categories did not differ significantly between the two groups (*P* = 0.236). The levels of FT3, FT4, T3, and T4 were lower, whereas the levels of TSH and TRAb were higher, in participants with thyroid function alterations (all *P* < 0.05). No significant differences were observed in TPOAb or TGAb levels between the two groups. Participants with thyroid function alterations also had lower TP and higher CRP levels (both *P* < 0.05).

### 3.2 Associations between serum 25(OH)D levels and thyroid function indicators

The associations between serum 25(OH)D levels and thyroid function indicators were examined using multiple linear regression models (**Table 2**). In the fully adjusted Model D, continuous ln[25(OH)D] was positively associated with FT4 (*β* = 0.119, 95% CI 0.012 to 0.226, *P* = 0.030, *q* = 0.030), FT3 (*β* = 0.190, 95% CI 0.088 to 0.291, *P* < 0.001, *q* < 0.001), T3 (*β* = 0.269, 95% CI 0.148 to 0.389, *P* < 0.001, *q* < 0.001), and T4 (*β* = 0.244, 95% CI 0.076 to 0.411, *P* = 0.005, *q* = 0.008), and was inversely associated with TSH (*β* = −0.608, 95% CI −1.055 to −0.161, *P* = 0.008, *q* = 0.010). When 25(OH)D was analyzed as a categorical variable, participants with levels <20 ng/mL had lower T3 levels than those with levels ≥30 ng/mL (β = −0.388, 95% CI −0.583 to −0.194, *P* < 0.001, *q* = 0.001). No other categorical associations were statistically significant after correction for multiple comparisons.

**Table 2:** Associations of continuous or categorical serum 25(OH)D levels with thyroid function indicators by multiple linear regression models.

Variable	Model A			Model B			Model C			Model D		
	β (95% CI)	P-value	q-value	β (95% CI)	P-value	q-value	β (95% CI)	P-value	q-value	β (95% CI)	P-value	q-value
FT4												
Continuous ln[25(OH)D]	0.077 (-0.027, 0.180)	0.145	0.145	0.095 (-0.009, 0.199)	0.074	0.074	0.098 (-0.008, 0.204)	0.070	0.070	0.119 (0.012, 0.226)	0.030	0.030
≥30 ng/mL	Reference			Reference			Reference			Reference		
20–<30 ng/mL	-0.048 (-0.227, 0.131)	0.598	0.929	-0.040 (-0.220, 0.139)	0.657	0.975	-0.034 (-0.214, 0.146)	0.710	0.933	-0.026 (-0.202, 0.151)	0.774	0.959
<20 ng/mL	-0.050 (-0.225, 0.125)	0.571	0.929	-0.058 (-0.234, 0.118)	0.515	0.975	-0.052 (-0.229, 0.124)	0.559	0.933	-0.054 (-0.227, 0.120)	0.544	0.959
FT3												
Continuous ln[25(OH)D]	0.157 (0.056, 0.258)	0.003	0.006	0.174 (0.073, 0.275)	<0.001	0.002	0.173 (0.071, 0.276)	0.001	0.003	0.190 (0.088, 0.291)	<0.001	<0.001
≥30 ng/mL	Reference			Reference			Reference			Reference		
20–<30 ng/mL	-0.032 (-0.210, 0.145)	0.720	0.929	-0.027 (-0.202, 0.149)	0.764	0.975	-0.025 (-0.202, 0.151)	0.777	0.933	-0.011 (-0.181, 0.159)	0.899	0.959
<20 ng/mL	-0.092 (-0.266, 0.082)	0.297	0.809	-0.095 (-0.267, 0.077)	0.277	0.786	-0.093 (-0.267, 0.080)	0.289	0.722	-0.087 (-0.255, 0.080)	0.306	0.766
TSH												
Continuous ln[25(OH)D]	-0.390 (-0.813, 0.034)	0.071	0.089	-0.458 (-0.885, -0.031)	0.036	0.045	-0.495 (-0.929, -0.061)	0.026	0.032	-0.608 (-1.055, -0.161)	0.008	0.010
≥30 ng/mL	Reference			Reference			Reference			Reference		
20–<30 ng/mL	0.094 (-0.639, 0.828)	0.800	0.929	0.067 (-0.669, 0.804)	0.857	0.975	0.044 (-0.697, 0.784)	0.908	0.933	0.070 (-0.673, 0.813)	0.853	0.959
<20 ng/mL	-0.032 (-0.750, 0.685)	0.929	0.929	-0.011 (-0.734, 0.711)	0.975	0.975	-0.031 (-0.757, 0.695)	0.933	0.933	0.019 (-0.712, 0.750)	0.959	0.959
T3												
Continuous ln[25(OH)D]	0.272 (0.153, 0.390)	<0.001	<0.001	0.286 (0.170, 0.402)	<0.001	<0.001	0.275 (0.158, 0.391)	<0.001	<0.001	0.269 (0.148, 0.389)	<0.001	<0.001
≥30 ng/mL	Reference			Reference			Reference			Reference		
20–<30 ng/mL	-0.221 (-0.426, -0.017)	0.034	0.170	-0.207 (-0.407, -0.007)	0.043	0.215	-0.227 (-0.425, -0.029)	0.024	0.122	-0.216 (-0.413, -0.018)	0.032	0.162
<20 ng/mL	-0.396 (-0.596, -0.196)	<0.001	0.001	-0.386 (-0.583, -0.190)	<0.001	0.001	-0.403 (-0.596, -0.209)	<0.001	<0.001	-0.388 (-0.583, -0.194)	<0.001	0.001
T4												
Continuous ln[25(OH)D]	0.197 (0.040, 0.355)	0.015	0.024	0.222 (0.063, 0.381)	0.006	0.011	0.223 (0.061, 0.385)	0.007	0.012	0.244 (0.076, 0.411)	0.005	0.008
≥30 ng/mL	Reference			Reference			Reference			Reference		
20–<30 ng/mL	-0.025 (-0.299, 0.249)	0.858	0.929	-0.009 (-0.284, 0.266)	0.946	0.975	-0.022 (-0.298, 0.254)	0.874	0.933	-0.020 (-0.297, 0.258)	0.887	0.959
<20 ng/mL	-0.135 (-0.403, 0.134)	0.324	0.809	-0.138 (-0.408, 0.132)	0.315	0.786	-0.149 (-0.420, 0.122)	0.279	0.722	-0.152 (-0.425, 0.121)	0.275	0.766

25(OH)D, 25-hydroxyvitamin D; FT3, free triiodothyronine; FT4, free thyroxine; TSH, thyroid-stimulating hormone; T3, triiodothyronine; T4, thyroxine. Model A was unadjusted; Model B adjusted for age, sex, and BMI; Model C further adjusted for smoking status and season of blood draw; and Model D further adjusted for TP and CRP. *P* values were corrected for multiple comparisons using the Benjamini–Hochberg method, and *q* values represent the adjusted *P* values.

Moreover, we examined continuous and categorical 25(OH)D levels in relation to thyroid autoantibodies and thyroid hormone sensitivity indices. In the fully adjusted Model D, continuous ln[25(OH)D] was not significantly associated with TRAb (*β* = 0.042, 95% CI −0.209 to 0.293), TGAb (*β* = 0.339, 95% CI −0.290 to 0.969), or TPOAb (*β* = −0.246, 95% CI −0.890 to 0.397). Similarly, no significant associations were observed with TFQI (*β* = −0.063, 95% CI −0.155 to 0.028), TSHI (*β* = −0.196, 95% CI −0.559 to 0.166), or TT4RI (*β* = −14.580, 95% CI −40.614 to 11.454). None of these associations were statistically significant after correction for multiple comparisons, and no significant associations were observed in the categorical analyses (all *q* > 0.05). Full estimates are provided in **Supplementary Tables S3** and **S4**.

### 3.3 Association between serum 25(OH)D levels and thyroid function alterations

We examined the associations between continuous and categorical 25(OH)D levels and thyroid function alterations using logistic regression models (**Table 3**). Continuous ln[25(OH)D] was inversely associated with the odds of thyroid function alterations in Models A–C. In Model C, a one-unit increase in ln[25(OH)D] was associated with lower odds of thyroid function alterations (OR = 0.566, 95% CI 0.317–0.988, *P* = 0.048). After further adjustment for TP and CRP in Model D, the association remained inverse but was attenuated and was no longer statistically significant (OR = 0.569, 95% CI 0.309–1.021, *P* = 0.063). In the categorical analysis, compared with participants with 25(OH)D levels ≥30 ng/mL, no statistically significant associations were observed for either the 20–<30 ng/mL or <20 ng/mL categories across Models A–D.

**Table 3:** Associations of continuous or categorical serum 25(OH)D levels with thyroid function alterations by logistic regression models.

Variable	Model A		Model B		Model C		Model D	
	OR (95% CI)	P-value	OR (95% CI)	P-value	OR (95% CI)	P-value	OR (95% CI)	P-value
Continuous ln[25(OH)D]	0.537 (0.306–0.920)	0.026	0.527 (0.298–0.912)	0.024	0.566 (0.317–0.988)	0.048	0.569 (0.309–1.021)	0.063
≥30 ng/mL	Reference		Reference		Reference		Reference	
20–<30 ng/mL	1.431 (0.554–4.030)	0.473	1.458 (0.561–4.129)	0.454	1.427 (0.543–4.083)	0.484	1.321 (0.485–3.915)	0.597
<20 ng/mL	2.030 (0.808–5.603)	0.146	2.065 (0.814–5.748)	0.141	2.031 (0.792–5.702)	0.154	1.959 (0.739–5.713)	0.192

25(OH)D, 25-hydroxyvitamin D; OR, odds ratio; CI, confidence interval; BMI, body mass index; TP, total protein; CRP, C-reactive protein. Continuous 25(OH)D was natural log-transformed. Model A was unadjusted; Model B adjusted for age, sex, and BMI; Model C further adjusted for smoking status and season of blood draw, and Model D further adjusted for TP and CRP. For categorical analyses, ≥30 ng/mL was the reference group.

In the sensitivity analysis excluding participants with isolated low FT3, the inverse association between continuous ln[25(OH)D] and thyroid function alterations remained statistically significant in Models A and B, but was attenuated after further adjustment in Models C and D. In Model D, the OR was 0.579 (95% CI 0.306–1.075, *P* = 0.087). No significant associations were observed for the categorical 25(OH)D analyses. Detailed results are provided in **Supplementary Table S5**.

In subgroup analyses based on continuous ln[25(OH)D], an inverse association was observed in men (OR = 0.155, 95% CI 0.042–0.470, *P* = 0.002), but not in women (OR = 1.113, 95% CI 0.508–2.466, *P* = 0.789), with a significant interaction by sex (*P* for interaction = 0.017). Similarly, an inverse association was observed among participants aged ≥65 years (OR = 0.275, 95% CI 0.099–0.671, *P* = 0.008), but not among those aged <65 years (OR = 1.276, 95% CI 0.522–3.183, *P* = 0.592), with a significant interaction by age group (*P* for interaction = 0.029) (**Figure 1**; **Supplementary Table S6**).

**Figure 1:**
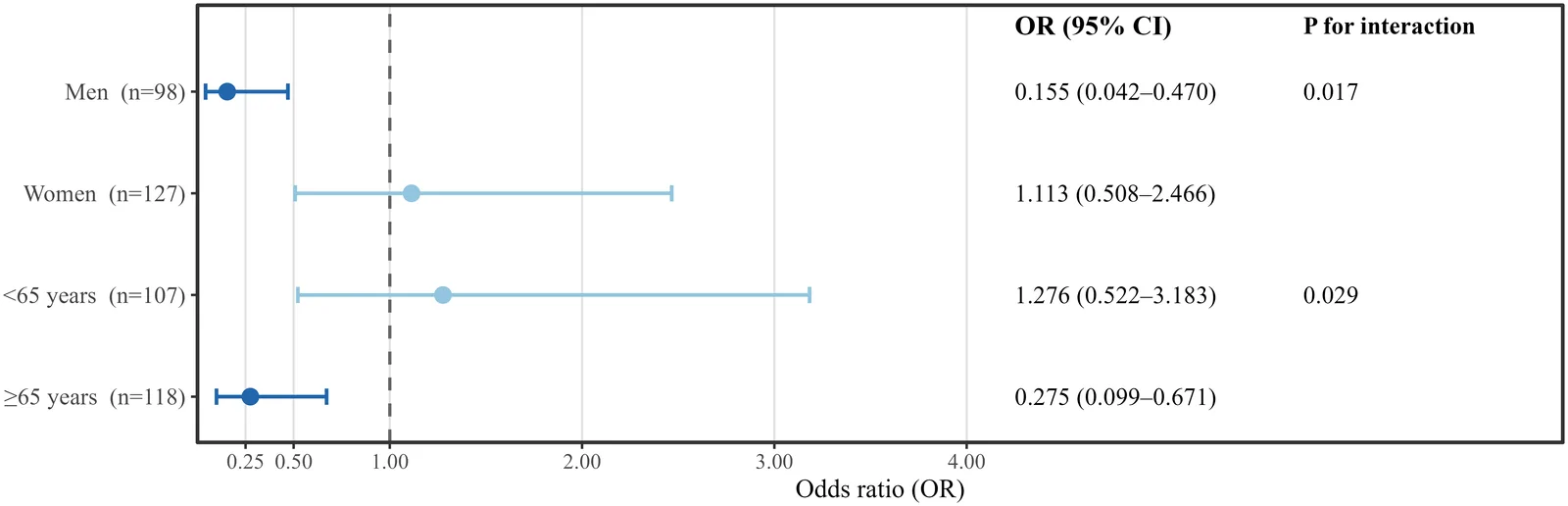
Associations of serum 25(OH)D with thyroid function alterations in subgroup analyses. 25(OH)D, 25-hydroxyvitamin D; OR, odds ratio; CI, confidence interval. Subgroup analyses were based on continuous ln[25(OH)D] and adjusted according to Model D, with adjustment for age, sex, BMI, smoking status, season of blood draw, TP, and CRP, except for the corresponding stratification variable.

### 3.4 Restricted cubic spline analysis

We further explored the association between serum 25(OH)D levels and thyroid function alterations using restricted cubic spline analysis. The estimated curve showed a decreasing trend in the odds of thyroid function alterations with increasing serum 25(OH)D levels, using 20.0 ng/mL as the reference value (**Figure 2**). However, neither the overall association nor the nonlinear component was statistically significant (*P* overall = 0.239; *P* nonlinear = 0.953).

**Figure 2:**
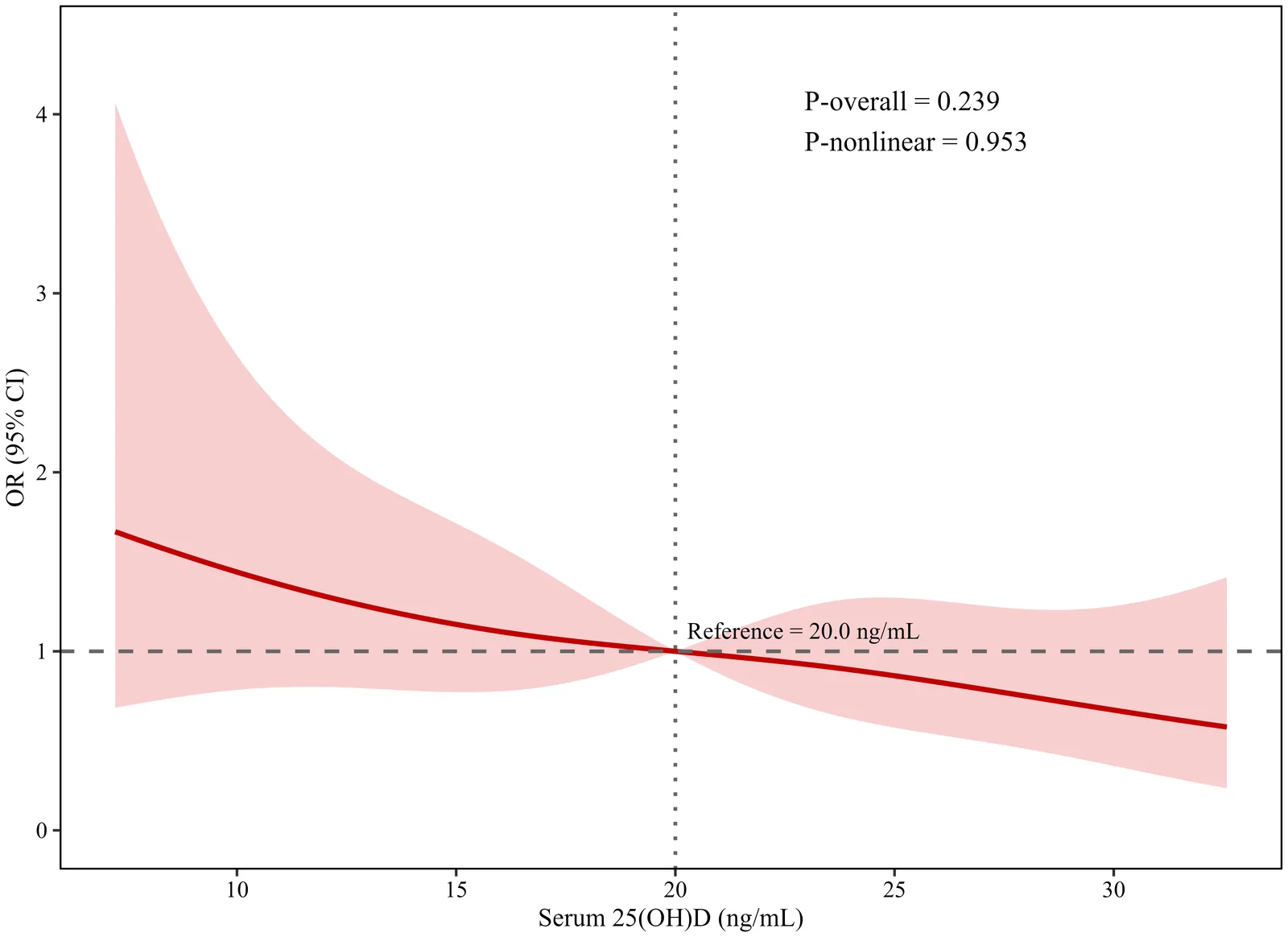
Restricted cubic spline analysis of the association between serum 25(OH)D levels and thyroid function alterations. The model was adjusted for age, sex, BMI, smoking status, season of blood draw, TP, and CRP. The reference value was 20.0 ng/mL. 25(OH)D, 25-hydroxyvitamin D; OR, odds ratio; CI, confidence interval; BMI, body mass index; TP, total protein; CRP, C-reactive protein. *P* overall = 0.239; *P* nonlinear = 0.953.

### 3.5 Exploratory LASSO logistic regression analysis

As an exploratory analysis, we applied LASSO logistic regression to examine the contribution of serum 25(OH)D together with other candidate variables to thyroid function alterations. The model included age, sex, BMI, season, smoking status, GLU, TP, CRP, and continuous 25(OH)D as candidate variables. The optimal penalty parameter selected by 10-fold cross-validation yielded a cross-validated AUROC of 0.64, indicating modest discriminative performance. At the selected penalty value, CRP, GLU, 25(OH)D, TP, and high sunlight season had nonzero coefficients. The coefficient for 25(OH)D was negative (−0.221). Detailed coefficients are provided in **Supplementary Table S7**.

## 4 Discussion

This study evaluated the relationship between serum 25(OH)D levels and thyroid function alterations and multiple thyroid function indicators in hospitalized adults. A total of 225 adult inpatients were included, and 91 (40.4%) had thyroid function alterations. Compared with participants with normal thyroid function, participants with thyroid function alterations had lower 25(OH)D levels, lower levels of FT3, FT4, T3, and T4, and higher levels of TSH and TRAb. In the multivariable linear regression models, serum 25(OH)D was positively associated with FT4, FT3, T3, and T4 and inversely associated with TSH. For thyroid function alterations, the inverse association with continuous ln[25(OH)D] was statistically significant in Models A–C but was attenuated after further adjustment for TP and CRP in Model D. A similar attenuation was observed after excluding participants with isolated low FT3. Subgroup analyses showed significant interactions by both sex and age group, with inverse associations observed in men and in participants aged ≥65 years. Exploratory LASSO analysis further retained 25(OH)D together with CRP, GLU, TP, and season among the selected variables.

Firstly, serum 25(OH)D concentrations were generally low in this inpatient population. In our study, the median 25(OH)D levels were 21.4 ng/mL in participants with normal thyroid function and 18.0 ng/mL in those with thyroid function alterations. This distribution was broadly consistent with previous hospital based studies reporting a high prevalence of low vitamin D status among inpatients (29, 30). Several factors may contribute to lower circulating 25(OH)D levels in hospitalized adults, including reduced sunlight exposure, inadequate dietary intake, older age, and underlying illness (30–35). Importantly, acute inflammatory illness may also reduce measured serum 25(OH)D concentrations because 25(OH)D can behave as a negative acute phase reactant (36, 37). Therefore, the relatively low 25(OH)D levels observed in our study may reflect not only nutritional and lifestyle factors, but also the clinical condition of hospitalized participants.

In our study, we further found that serum 25(OH)D was positively associated with FT4, FT3, T3, and T4 and inversely associated with TSH. Because total T3 and T4 concentrations are influenced by circulating binding proteins (38), TP was included in Model D as an indicator of protein status. The associations of 25(OH)D with T3 and T4 remained statistically significant after this adjustment. A hospital based study of adults with type 2 diabetes similarly reported higher FT3 levels in participants with sufficient vitamin D status (39), while Zhang et al. reported an inverse association between 25(OH)D and TSH in middle aged and elderly men (17). Several mechanisms may help explain these findings. Vitamin D receptor and 1α-hydroxylase have been identified in thyroid tissue (16), and vitamin D may also influence peripheral thyroid hormone metabolism through deiodinases, particularly Dio2, which is involved in the conversion of T4 to T3 in target tissues (14). In a diabetic rat model, vitamin D3 supplementation improved the thyroid hormone profile and was accompanied by increased Dio2 expression (40), which may partly explain the positive associations observed with FT3 and T3. The concurrent positive association with FT4 and inverse association with TSH further suggest that the relationship between 25(OH)D and thyroid function may involve multiple components of thyroid hormone regulation rather than peripheral T4-to-T3 conversion alone (14, 17). However, no significant associations were observed between 25(OH)D and TPOAb, TGAb, TRAb, or the thyroid hormone sensitivity indices TFQI, TSHI, and TT4RI. Similarly, an analysis of 8, 126 adults from NHANES 2007–2012 found no linear associations of serum vitamin D with TSHI, TFQI, or TT4RI after multivariable adjustment (41). These results suggest that the association between 25(OH)D and thyroid function was mainly reflected in circulating thyroid hormone levels rather than in thyroid autoantibodies or thyroid hormone sensitivity indices. We therefore further examined its association with overall thyroid function alterations.

In logistic regression analyses, higher serum 25(OH)D levels were associated with lower odds of thyroid function alterations in Models A–C, although the association was attenuated after further adjustment for TP and CRP in Model D. In contrast, the categorical analyses did not show statistically significant associations, and no clear graded relationship was observed across the three 25(OH)D categories. The inverse association observed in the continuous analyses is broadly consistent with previous human studies suggesting that lower vitamin D status is associated with hypothyroid conditions and abnormal thyroid function. In a community based program, Mirhosseini et al. (18) reported that higher serum 25(OH)D concentrations were associated with a lower risk of hypothyroidism. Similarly, Kim et al. (42) and Appunni et al. (43) reported associations between low vitamin D status and hypothyroid conditions in clinical and population based studies. More recently, Mendelian randomization analysis (44) suggested that genetically predicted higher serum 25(OH)D concentrations were associated with lower risks of high TSH and autoimmune hypothyroidism.

However, thyroid function abnormalities in hospitalized patients should also be interpreted in the context of nonthyroidal illness syndrome (NTIS). NTIS is typically characterized by reduced circulating T3 or FT3, while more severe or prolonged systemic illness may also be accompanied by reductions in T4 and alterations in TSH (9, 10, 45, 46). These alterations may involve changes in deiodinase activity, inflammatory signaling, nutritional status, and suppression of the hypothalamic–pituitary–thyroid axis rather than primary thyroid disease (10, 45, 46). A meta-analysis of critically ill adults also found that lower T3/FT3 and FT4 levels were associated with poorer clinical outcomes (47). In our cohort, isolated low FT3 accounted for 11 of the 91 participants with thyroid function alterations. After excluding these participants, the association with ln[25(OH)D] remained inverse but was no longer statistically significant in the fully adjusted model. Therefore, isolated low FT3 may not fully explain the observed association, although illness-related thyroid changes remain a potential source of residual confounding (9, 45).

The RCS analysis showed a decreasing trend in the odds of thyroid function alterations with increasing serum 25(OH)D levels, but neither the overall association nor the nonlinear component was statistically significant. Previous spline based studies have reported heterogeneous dose response patterns between vitamin D and thyroid related outcomes. An NHANES analysis found nonlinear associations of serum vitamin D with TFQI and FT3/FT4, while no nonlinear relationships were identified for TSHI or TT4RI (41). A recent prospective cohort study also reported a nonlinear association between circulating vitamin D and hypothyroidism (48). These findings suggest that the shape of the association may differ according to the population and thyroid related outcome examined. In our exploratory LASSO analysis, 25(OH)D was retained together with CRP, fasting glucose, total protein, and season, suggesting that 25(OH)D remained one of several selected variables when inflammatory, metabolic, nutritional, and seasonal factors were considered.

This study has several limitations. First, the cross-sectional design precludes causal inference and cannot determine the temporal direction of the association between serum 25(OH)D and thyroid function alterations. Reverse causation is also possible because systemic illness may affect both 25(OH)D levels and thyroid function tests. Second, the relatively small sample size may have limited statistical power. Third, residual confounding cannot be excluded, particularly for iodine status, detailed medication exposure, vitamin D supplementation, underlying disease type, and illness severity in this clinically heterogeneous inpatient population. The exploratory LASSO model also showed modest discrimination and lacked external validation. Finally, the specific indications for ordering both tests were not consistently available, and the single-center sample included only inpatients who underwent both 25(OH)D and complete thyroid function testing, which may have introduced selection bias and limited generalizability.

## 5 Conclusion

In conclusion, serum 25(OH)D was associated with multiple thyroid function indicators in hospitalized adults, including positive associations with FT4, FT3, T3, and T4 and an inverse association with TSH. An inverse association with thyroid function alterations was observed in Models A–C but was attenuated after further adjustment for TP and CRP. Given the cross-sectional design and clinical heterogeneity of hospitalized patients, prospective studies are needed to clarify the temporal direction and clinical significance of these associations.

## Data Availability

The data analyzed in this study is subject to the following licenses/restrictions: The datasets analyzed during the current study are not publicly available due to privacy restrictions on de-identified clinical data, but are available from the corresponding author on reasonable request and with permission from the First Affiliated Hospital of Xiamen University. Requests to access the datasets should be directed to Shaojie Liu, shaojieliu@xmu.edu.cn.
