# Supplementary material for: Association between serum 25-hydroxyvitamin D levels and thyroid function alterations in hospitalized adults: a retrospective cross-sectional study
Source: Front Endocrinol (Lausanne). 2026 Oct 14;17:1959544. doi: 10.3389/fendo.2026.1959544 (PMC13713329; doi:10.3389/fendo.2026.1959544)
Supplement: Supplementary file 1 [file Table1.docx]

**Supplementary materials**

Table S1. Major disease categories among hospitalized participants with available principal diagnosis information

Table S2. Biochemical patterns among participants with thyroid function alterations (n = 91)

Table S3. Associations of continuous or categorical serum 25(OH)D levels with thyroid autoantibodies by multiple linear regression models

Table S4. Associations of continuous or categorical serum 25(OH)D levels with thyroid hormone sensitivity indices by multiple linear regression models

Table S5. Sensitivity analysis of the association between serum 25(OH)D levels and thyroid function alterations after excluding participants with isolated low FT3

Table S6. Subgroup analyses of the association between continuous ln[25(OH)D] and thyroid function alterations

Table S7. Coefficients retained in the exploratory LASSO logistic regression analysis

**Table S1. Major disease categories among hospitalized participants with available principal diagnosis information**

| **Major disease category** | **n** | **% of participants** |
| --- | --- | --- |
| Cardiovascular | 40 | 18.8% |
| Musculoskeletal/rheumatic | 38 | 17.8% |
| Neurologic | 32 | 15.0% |
| Renal/urologic | 30 | 14.1% |
| Respiratory/infectious | 22 | 10.3% |
| Other diseases | 51 | 23.9% |
| Total | 213 | 100.0% |

**Note:** Principal diagnosis information was available for 213 of 225 participants (94.7%), while 12 participants (5.3%) had missing information. Percentages were calculated among participants with available principal diagnosis information.

**Table S2. Biochemical patterns among participants with thyroid function alterations (n = 91)**

| **Biochemical pattern** | **TSH** | **FT4** | **FT3** | **n (%)** |
| --- | --- | --- | --- | --- |
| Subclinical hypothyroidism | ↑ | – | – | 29 (31.9%) |
| Clinical hypothyroidism | ↑ | ↓ | ↓ | 10 (11.0%) |
|  | ↑ | ↓ | – | 6 (6.6%) |
| Isolated low FT3 | – | – | ↓ | 11 (12.1%) |
| Subclinical hyperthyroidism | ↓ | – | – | 7 (7.7%) |
| Clinical hyperthyroidism | ↓ | ↑ | ↑ | 6 (6.6%) |
|  | ↓ | ↑ | – | 1 (1.1%) |
| Other abnormal patterns | ↑ | – | ↓ | 3 (3.3%) |
|  | – | ↓ | – | 6 (6.6%) |
|  | – | ↓ | ↓ | 4 (4.4%) |
|  | – | ↑ | – | 4 (4.4%) |
|  | ↓ | ↓ | ↓ | 2 (2.2%) |
|  | ↓ | – | ↓ | 1 (1.1%) |
|  | – | – | ↑ | 1 (1.1%) |
| **Total** |  |  |  | **91 (100.0%)** |

**Note:** ↑, above the laboratory reference range; ↓, below the laboratory reference range; –, within the laboratory reference range. Abbreviations: FT3, free triiodothyronine; FT4, free thyroxine; TSH, thyroid-stimulating hormone.

**Table S3. Associations of continuous or categorical serum 25(OH)D levels with thyroid autoantibodies by multiple linear regression models**

|  | **Model A** | | | **Model B** | | | **Model C** | | | **Model D** | | |
| --- | --- | --- | --- | --- | --- | --- | --- | --- | --- | --- | --- | --- |
|  | ***β (95% CI)*** | ***P-value*** | ***q-value*** | ***β (95% CI)*** | ***P-value*** | ***q-value*** | ***β (95% CI)*** | ***P-value*** | ***q-value*** | ***β (95% CI)*** | ***P-value*** | ***q-value*** |
| **TRAb** | | | | | | | | | | | | |
| Continuous ln[25(OH)D] | -0.027 (-0.268, 0.214) | 0.826 | 0.826 | 0.021 (-0.219, 0.261) | 0.864 | 0.864 | 0.065 (-0.178, 0.307) | 0.598 | 0.708 | 0.042 (-0.209, 0.293) | 0.744 | 0.744 |
| ≥30 ng/mL | Reference |  |  | Reference |  |  | Reference |  |  | Reference |  |  |
| 20–<30 ng/mL | -0.147 (-0.560, 0.267) | 0.486 | 0.972 | -0.173 (-0.582, 0.236) | 0.406 | 0.987 | -0.171 (-0.579, 0.238) | 0.411 | 0.986 | -0.171 (-0.580, 0.239) | 0.413 | 0.961 |
| <20 ng/mL | -0.002 (-0.407, 0.403) | 0.992 | 0.992 | -0.075 (-0.476, 0.326) | 0.713 | 0.987 | -0.076 (-0.477, 0.324) | 0.708 | 0.986 | -0.064 (-0.467, 0.339) | 0.755 | 0.961 |
| **TGAb** | | | | | | | | | | | | |
| Continuous ln[25(OH)D] | 0.330 (-0.261, 0.921) | 0.273 | 0.616 | 0.391 (-0.205, 0.986) | 0.198 | 0.593 | 0.336 (-0.270, 0.942) | 0.276 | 0.708 | 0.339 (-0.290, 0.969) | 0.290 | 0.677 |
| ≥30 ng/mL | Reference |  |  | Reference |  |  | Reference |  |  | Reference |  |  |
| 20–<30 ng/mL | 0.384 (-0.635, 1.403) | 0.459 | 0.972 | 0.337 (-0.682, 1.357) | 0.515 | 0.987 | 0.333 (-0.691, 1.357) | 0.522 | 0.986 | 0.333 (-0.698, 1.363) | 0.525 | 0.961 |
| <20 ng/mL | 0.201 (-0.796, 1.197) | 0.692 | 0.992 | 0.114 (-0.886, 1.114) | 0.822 | 0.987 | 0.115 (-0.889, 1.119) | 0.822 | 0.986 | 0.122 (-0.892, 1.137) | 0.812 | 0.961 |
| **TPOAb** | | | | | | | | | | | | |
| Continuous ln[25(OH)D] | -0.256 (-0.866, 0.355) | 0.410 | 0.616 | -0.187 (-0.800, 0.427) | 0.549 | 0.824 | -0.119 (-0.742, 0.505) | 0.708 | 0.708 | -0.246 (-0.890, 0.397) | 0.451 | 0.677 |
| ≥30 ng/mL | Reference |  |  | Reference |  |  | Reference |  |  | Reference |  |  |
| 20–<30 ng/mL | 0.094 (-0.956, 1.144) | 0.860 | 0.992 | 0.008 (-1.039, 1.056) | 0.988 | 0.988 | 0.004 (-1.047, 1.056) | 0.993 | 0.993 | 0.026 (-1.025, 1.078) | 0.961 | 0.961 |
| <20 ng/mL | 0.419 (-0.608, 1.446) | 0.422 | 0.972 | 0.269 (-0.758, 1.297) | 0.606 | 0.987 | 0.261 (-0.770, 1.292) | 0.618 | 0.986 | 0.330 (-0.705, 1.365) | 0.530 | 0.961 |

Note: Thyroid autoantibody levels were natural log-transformed before regression analysis. Continuous 25(OH)D was natural log-transformed, and ≥30 ng/mL was the reference group in categorical analyses. Model A was unadjusted; Model B adjusted for age, sex, and BMI; Model C further adjusted for smoking status and season of blood draw; and Model D further adjusted for TP and CRP. P values were adjusted using the Benjamini–Hochberg method separately within each model for continuous and categorical 25(OH)D analyses, and q values represent the adjusted P values. Abbreviations: 25(OH)D, 25-hydroxyvitamin D; BMI, body mass index; TP, total protein; CRP, C-reactive protein; TPOAb, thyroid peroxidase antibody; TGAb, thyroglobulin antibody; TRAb, thyrotropin receptor antibody.

**Table S4. Associations of continuous or categorical serum 25(OH)D levels with thyroid hormone sensitivity indices by multiple linear regression models**

|  | **Model A** | | | **Model B** | | | **Model C** | | | **Model D** | | |
| --- | --- | --- | --- | --- | --- | --- | --- | --- | --- | --- | --- | --- |
|  | ***β (95% CI)*** | ***P-value*** | ***q-value*** | ***β (95% CI)*** | ***P-value*** | ***q-value*** | ***β (95% CI)*** | ***P-value*** | ***q-value*** | ***β (95% CI)*** | ***P-value*** | ***q-value*** |
| **TFQI** | | | | | | | | | | | | |
| Continuous ln[25(OH)D] | -0.038 (-0.125, 0.049) | 0.389 | 0.707 | -0.038 (-0.127, 0.051) | 0.397 | 0.585 | -0.038 (-0.129, 0.052) | 0.406 | 0.520 | -0.063 (-0.155, 0.028) | 0.176 | 0.286 |
| ≥30 ng/mL | Reference |  |  | Reference |  |  | Reference |  |  | Reference |  |  |
| 20–<30 ng/mL | 0.005 (-0.146, 0.155) | 0.952 | 0.952 | 0.008 (-0.144, 0.159) | 0.922 | 0.985 | 0.011 (-0.142, 0.164) | 0.884 | 0.896 | 0.027 (-0.123, 0.177) | 0.725 | 0.974 |
| <20 ng/mL | -0.031 (-0.178, 0.116) | 0.676 | 0.952 | -0.030 (-0.179, 0.119) | 0.696 | 0.985 | -0.026 (-0.176, 0.124) | 0.730 | 0.896 | -0.007 (-0.155, 0.141) | 0.926 | 0.974 |
| **TSHI** | | | | | | | | | | | | |
| Continuous ln[25(OH)D] | -0.119 (-0.460, 0.221) | 0.491 | 0.707 | -0.121 (-0.468, 0.226) | 0.494 | 0.585 | -0.121 (-0.474, 0.232) | 0.500 | 0.520 | -0.196 (-0.559, 0.166) | 0.286 | 0.286 |
| ≥30 ng/mL | Reference |  |  | Reference |  |  | Reference |  |  | Reference |  |  |
| 20–<30 ng/mL | -0.322 (-0.905, 0.262) | 0.279 | 0.836 | -0.329 (-0.918, 0.261) | 0.273 | 0.819 | -0.350 (-0.943, 0.243) | 0.246 | 0.738 | -0.310 (-0.901, 0.281) | 0.303 | 0.909 |
| <20 ng/mL | -0.456 (-1.027, 0.114) | 0.117 | 0.699 | -0.467 (-1.045, 0.112) | 0.113 | 0.680 | -0.486 (-1.067, 0.096) | 0.101 | 0.607 | -0.435 (-1.016, 0.147) | 0.142 | 0.853 |
| **TT4RI** | | | | | | | | | | | | |
| Continuous ln[25(OH)D] | -4.711 (-29.422, 20.000) | 0.707 | 0.707 | -6.950 (-32.010, 18.109) | 0.585 | 0.585 | -8.260 (-33.526, 17.005) | 0.520 | 0.520 | -14.580 (-40.614, 11.454) | 0.271 | 0.286 |
| ≥30 ng/mL | Reference |  |  | Reference |  |  | Reference |  |  | Reference |  |  |
| 20–<30 ng/mL | 11.173 (-31.277, 53.623) | 0.604 | 0.952 | 12.807 (-29.934, 55.548) | 0.555 | 0.985 | 9.178 (-33.423, 51.779) | 0.672 | 0.896 | 10.846 (-31.796, 53.488) | 0.617 | 0.974 |
| <20 ng/mL | -2.933 (-44.447, 38.582) | 0.889 | 0.952 | 0.391 (-41.535, 42.316) | 0.985 | 0.985 | -2.766 (-44.532, 39.000) | 0.896 | 0.896 | 0.690 (-41.264, 42.643) | 0.974 | 0.974 |

Note: Continuous 25(OH)D was natural log-transformed, and ≥30 ng/mL was the reference group in categorical analyses. Model A was unadjusted; Model B adjusted for age, sex, and BMI; Model C further adjusted for smoking status and season of blood draw; and Model D further adjusted for TP and CRP. P values were adjusted using the Benjamini–Hochberg method separately within each model for continuous and categorical 25(OH)D analyses, and q values represent the adjusted P values. Abbreviations: 25(OH)D, 25-hydroxyvitamin D; BMI, body mass index; TP, total protein; CRP, C-reactive protein; TT4RI, thyrotroph T4 resistance index; TSHI, thyroid-stimulating hormone index; TFQI, thyroid feedback quantile-based index.

**Table S5. Sensitivity analysis of the association between serum 25(OH)D levels and thyroid function alterations after excluding participants with isolated low FT3**

| **Exposure** | **Model A** | | **Model B** | | **Model C** | | **Model D** | |
| --- | --- | --- | --- | --- | --- | --- | --- | --- |
|  | **OR (95% CI)** | **P-value** | **OR (95% CI)** | **P-value** | **OR (95% CI)** | **P-value** | **OR (95% CI)** | **P-value** |
|  |  |  |  |  |  |  |  |  |
| Continuous ln[25(OH)D] | 0.528 (0.294–0.929) | 0.029 | 0.517 (0.285–0.918) | 0.026 | 0.558 (0.305–1.003) | 0.054 | 0.579 (0.306–1.075) | 0.087 |
| ≥30 ng/mL | Reference |  | Reference |  | Reference |  | Reference |  |
| 20–<30 ng/mL | 1.171 (0.447–3.333) | 0.755 | 1.181 (0.448–3.376) | 0.744 | 1.166 (0.438–3.364) | 0.765 | 1.127 (0.411–3.347) | 0.820 |
| <20 ng/mL | 1.831 (0.725–5.072) | 0.217 | 1.850 (0.725–5.167) | 0.213 | 1.809 (0.703–5.097) | 0.235 | 1.730 (0.650–5.038) | 0.288 |

Note: This sensitivity analysis excluded 11 participants with isolated low FT3, leaving 214 participants for analysis. Continuous 25(OH)D was natural log-transformed, and ≥30 ng/mL was the reference group in categorical analyses. Model A was unadjusted; Model B adjusted for age, sex, and BMI; Model C further adjusted for smoking status and season of blood draw; and Model D further adjusted for TP and CRP. Abbreviations: 25(OH)D, 25-hydroxyvitamin D; FT3, free triiodothyronine; OR, odds ratio; CI, confidence interval; BMI, body mass index; TP, total protein; CRP, C-reactive protein.

**Table S6.** **Subgroup analyses of the association between continuous ln[25(OH)D] and thyroid function alterations**

| **Subgroup** | **N** | **Cases** | **Controls** | **OR (95% CI)** | **P-value** | **P for interaction** |
| --- | --- | --- | --- | --- | --- | --- |
| Men | 98 | 42 | 56 | 0.155 (0.042–0.470) | 0.002 | 0.017 |
| Women | 127 | 49 | 78 | 1.113 (0.508–2.466) | 0.789 |  |
| <65 years | 107 | 41 | 66 | 1.276 (0.522–3.183) | 0.592 | 0.029 |
| ≥65 years | 118 | 50 | 68 | 0.275 (0.099–0.671) | 0.008 |  |

Note: Serum 25(OH)D was analyzed as a natural log-transformed continuous variable. Subgroup models were adjusted according to Model D, excluding the corresponding stratification variable. Interactions were tested using product terms. Abbreviations: 25(OH)D, 25-hydroxyvitamin D; OR, odds ratio; CI, confidence interval; BMI, body mass index; TP, total protein; CRP, C-reactive protein.

**Table S7. Coefficients retained in the exploratory LASSO logistic regression analysis**

| Predictor | Coefficient |
| --- | --- |
| CRP | 0.350 |
| GLU | 0.280 |
| 25(OH)D | -0.221 |
| TP | -0.188 |
| High-sunlight season | -0.130 |

Note: LASSO penalized logistic regression was performed using 10-fold cross-validation. Candidate predictors included age, sex, BMI, smoking status, season, GLU, TP, CRP, and continuous 25(OH)D. Variables shown are those with nonzero coefficients at the selected penalty parameter. The analysis was exploratory, with a cross-validated AUROC of 0.64. Abbreviations: CRP, C-reactive protein; GLU, fasting plasma glucose; 25(OH)D, 25-hydroxyvitamin D; TP, total protein; BMI, body mass index; AUROC, area under the receiver operating characteristic curve.
